# Single-cell sequencing of the substantia nigra reveals microglial activation in a model of MPTP

**DOI:** 10.3389/fnagi.2024.1390310

**Published:** 2024-06-17

**Authors:** Qing Liu, Ziyu Liu, Wenmeng Xie, Yibo Li, Hongfang Wang, Sanbing Zhang, Wenyu Wang, Jiaxin Hao, Dandan Geng, Jing Yang, Lei Wang

**Affiliations:** ^1^Department of Human Anatomy, Hebei Medical University, Shijiazhuang, Hebei, China; ^2^Department of Hand and Foot Surgery, The Third Hospital of Shijiazhuang, Shijiazhuang, Hebei, China; ^3^Neuroscience Research Center, Hebei Medical University, Shijiazhuang, Hebei, China; ^4^Hebei Key Laboratory of Neurodegenerative Disease Mechanism, Shijiazhuang, Hebei, China; ^5^Zhejiang Provincial Key Laboratory of Aging and Cancer Biology, Hangzhou Normal University, Hangzhou, Zhejiang, China; ^6^The Key Laboratory of Neural and Vascular Biology, Ministry of Education, Hebei Medical University, Shijiazhuang, Hebei, China

**Keywords:** Parkinson’s disease, single-nucleus sequencing, microglia, cell–cell communications, cellular states

## Abstract

**Background:**

N-methyl-4-phenyl-1,2,3,6-tetrahydropyridine (MPTP) is a neurotoxin widely used to induce PD models, but the effect of MPTP on the cells and genes of PD has not been fully elucidated.

**Methods:**

Single-nucleus RNA sequencing was performed in the Substantia Nigra (SN) of MPTP mice. UMAP analysis was used for the dimensionality reduction visualization of the SN in the MPTP mice. Known marker genes highly expressed genes in each cluster were used to annotate most clusters. Specific Differentially Expressed Genes (DEGs) and PD risk genes analysis were used to find MPTP-associated cells. GO, KEGG, PPI network, GSEA and CellChat analysis were used to reveal cell type-specific functional alterations and disruption of cell-cell communication networks. Subset reconstruction and pseudotime analysis were used to reveal the activation status of the cells, and to find the transcription factors with trajectory characterized.

**Results:**

Initially, we observed specific DEGs and PD risk genes enrichment in microglia. Next, We obtained the functional phenotype changes in microglia and found that IGF, AGRN and PTN pathways were reduced in MPTP mice. Finally, we analyzed the activation state of microglia and revealed a pro-inflammatory trajectory characterized by transcription factors Nfe2l2 and Runx1.

**Conclusion:**

Our work revealed alterations in microglia function, signaling pathways and key genes in the SN of MPTP mice.

## 1 Introduction

Parkinson’s Disease (PD) is a common degenerative neurological disorder characterized by static tremor, bradykinesia, myotonia, and postural gait disorders (Bloem et al., 2021). The main pathological changes were the aggregation of α-synuclein and the progressive loss of Dopaminergic Neurons (DaNs) (Dauer and Przedborski, 2003). Globally, the majority of PD patients are aged over 65 years (Ou et al., 2021), and this proportion will gradually increase with the development of population aging, reaching 9.3 million people in 2030 (Dorsey et al., 2007). This will cause great economic and psychological burdens to society and families. Therefore, exploring the pathogenesis of PD and finding effective prevention targets are the key issues need to be solved.

Cell heterogeneity is important to studying the pathogenesis and pathological mechanism of disease (Armand et al., 2021). Traditional studies are usually conducted in tissue and can only examine the average expression of genes. Single-cell/nucleus RNA sequencing (sc/snRNA-seq) can detect gene expression in single cells. It is widely used to reveal the expression changes of specific genes of various cells in complex tissues under various physiological and pathological conditions (Booth et al., 2017). Although the progressive loss of DaNs is a major pathological change in PD, other types of cells, such as astrocytes and microglia are involved in PD (Maiti et al., 2017; Habib et al., 2020). A novel subtype of Alzheimer’s disease-associated microglia has been identified by using scRNA-seq. Abnormal expression of genes associated with lipid metabolic pathways and phagocytosis affects the clearance of amyloid-beta plaques (Keren-Shaul et al., 2017). In addition, snRNA-seq results in patients with idiopathic PD showed that microglia and astrocytes are associated with inflammatory signaling and immunomodulatory effects in PD (Smajić et al., 2022). Therefore, it is particularly critical to identify cell types and molecular mechanisms related to PD pathology.

N-methyl-4-phenyl-1,2,3,6-tetrahydropyridine (MPTP), a lipophilic prototoxin commonly used to induce PD models in rodents and primates (Tang et al., 2023). MPTP can cross the blood–brain barrier and be oxidized to 1-methyl-4-phenylpyridinium (MPP) by monoamine oxidase B, and then MPP is concentrated in the dopaminergic terminals and cell bodies by the dopamine uptake transporter to produce toxicity (Jackson-Lewis and Przedborski, 2007). This process often accompanied by changes in different cells. For example, DaNs damage (Lin et al., 2023), microglial inflammation (Pike et al., 2022), astrogliosis (Hou et al., 2017). But how MPTP affects cell state at the single-cell transcriptional level remains to be further elucidated.

Here, we applied snRNA-seq to study complex cell state changes in the Substantia Nigra (SN) of MPTP model mice and matched control mice. First, based on the number of specific Differentially Expressed Genes (DEGs) and PD risk genes enrichment analysis, it was found that the transcript changes in microglia were the most significant. The function of microglia was revealed by GO, KEGG, and GSEA analysis of microglia-specific DEGs. Second, we analyzed the changes in the communication relationship between microglia and other cells to explore the effects of the communication pattern of microglia in MPTP. Eventually, the activation states of microglia were characterized by trajectory reconstruction analysis, and the key transcription factors Nfe2l2 and Runx1 that may mediate microglia activation were discovered. Altogether, our work will provide evidence to elucidate the cellular heterogeneity in SN and the complex molecular mechanisms in PD. This result will have important implications for the treatment of PD and other neurodegenerative diseases in the future.

## 2 Materials and methods

### 2.1 Ethics statement

All animal experiments in this study were approved by the Animal Ethics and Welfare Committee of Hebei Medical University (IACUC-Hebmu-P2022153). All procedures were conducted following the guidelines of the animal ethical and welfare committee. All applicable institutional and/or national guidelines for the care and use of animals were followed.

### 2.2 Tissue dissection

Eight ten-week-old male mice on the ICR/JCL background were purchased from Beijing Vital River Laboratory Animal Technology Corporation, Beijing, China. The animals were exposed to 12 h of light/12 h of darkness under specific pathogen-free conditions at 24 ± 2°C, allowing them to freely obtain food and water. MPTP (Macklin Biochemical, Shanghai, China) and physiological saline were intraperitoneally injected at a dose of 30 mg/kg/day for 5 consecutive days to prepare MPTP-induced PD model mice (MPTP) and control group model mice (Con) 7 days after the last injection, behavioral tests were performed (Supplementary Figure S1A). After completion of the test, the mice were anesthetized with pentobarbital sodium (50 mg/kg, intraperitoneal injection) and sacrificed by cervical dislocation. The anatomical coordinates of the SN were 3.00 mm posterior to the anterior fontanel, 1.1 mm lateral to the midline and 4.5 mm subdural. The SN was cut at a coronal section 3–4 mm behind the anterior fontanelle (posterior to the hippocampal region), about 2–3 mm thick. The color of the SN is different from other brain regions, and it has relatively clear boundaries, which can be picked up with tweezers. SN was quickly frozen in liquid nitrogen after remove.

### 2.3 Behavioral tests

Open field test. The field test was used to determine the spontaneous exploratory activity of the mice. The mice were put into the testing room and habituated the day before the experiment. The mice were placed in the center of the open field, and their movement was monitored (videotaped) for 5 min. Videos were analyzed in terms of the following parameters: total distance, and average speed (Supplementary Figures S1B,C).

#### 2.3.1 Rotatod rod test

The mice were placed on an accelerating rotatod. During behavioral training, the rotation speed of the rotator remained at 10 rpm for 5 min. During the formal test, the rotator speed was initially set at 4 rpm and then gradually increased to 40 rpm over 2 min, and the time for the animal to drop was recorded (Supplementary Figure S1D).

#### 2.3.2 Climbing test

We made a straight wooden pole with a diameter of 0.8 cm and a height of 60 cm. There was a small wooden ball on the top of the pole, and gauze was applied to prevent the mice from slipping. A mouse was placed on a vertical wooden pole with its head facing upward, and the time it took to climb to the bottom of the pole was measured. Each test interval was more than 3 min, and the average value was taken 3 times (Supplementary Figures S1E–G).

#### 2.3.3 Treadmill test

The exercise speed was running at 2 m/min for 5 min, at 3 m/min for 5 min, and then at 5 m/min for 20 min with no inclination. The distance and speed for the animal to drop were recorded (Supplementary Figures S1H,I).

### 2.4 Sample preparation for nuclei isolation

Four samples from each group were pooled for nuclei isolated according to the nuclear isolation by snRNA-seq protocol of 10× Genomics (Shbio, #52009–10, Shanghai, China). In brief, the tissue was lysed in chilled lysis buffer (10 mM Tris HCl, 10 mM NaCl, 3 mM MgCl2, 0.1% Nonidet P40). Then, the suspension was filtered, and the nuclei were pelleted by centrifugation. Nuclei pellets were then washed in nuclei wash and resuspension buffer, [1× PBS (phosphate buffered saline), 1% BSA (bovine serum albumin), 0.2 U/μl RNase inhibitor], filtered and pelleted again. Nuclei pellets were resuspended again, and 5 μL of the nuclear suspension was taken for cell nuclei counting by trypan blue staining and the concentration was adjusted according to subsequent experiments (Supplementary Table S1).

### 2.5 Library construction and sequencing

Sorted nuclei were processed using the 10× Chromium Next GEM Single Cell 3’ Kit Library & Gel Bear Kit v3.1 (10× Genomics, 1,000,121) to generate cDNA libraries. The quality of cDNA was assessed using the Agilent 2,100 Bioanalyzer System. Sequencing was performed on an Illumina NovaSeq 6,000-S2.

### 2.6 Data demultiplexing and quality control

We used Cell Ranger 5.0.1 (10× Genomics) to process raw sequencing data, and Seurat (version 4.0) was applied for downstream analysis. Before we started the downstream analysis, we focused on four filtering metrics to guarantee the reliability of our data. (1) Cells with detected genes out of the range of 200–5,000 were removed; (2) cells with Unique Molecular Identifiers (UMIs) greater than 10,000 were removed to filter out the doublet-like cells; (3) nuclei with a percentage of expressed mitochondrial genes greater than 0.2 were removed to rule out apoptotic cells, as well as ribosomal genes greater than 0.01 and dissociated genes greater than 0.015 (van den Brink et al., 2017; Guo et al., 2021a); (4) genes detected in fewer than three cells were filtered to avoid cellular stochastic events. As a result, 22,983 nuclei remained for downstream analysis (Supplementary Figure S2).

### 2.7 Multi-sample integration analysis and PCA dimensionality reduction

For each sample, the top 2,000 most variable genes were identified based on the mean and dispersion (variance/mean) of all genes for later integration analysis. The top 20 PCs were taken for subsequent cluster analysis. The calculation method of dispersion values for each gene was calculated according to the article (Zheng et al., 2017).

### 2.8 Clustering and cell annotation

To determine the cell types that make up the SN in mice, the Louvain algorithm was used to cluster the normalized data and obtain 25 clusters. Uniform Manifold Approximation and Projection (UMAP) was used for the final dimension reduction and visualization. The Wilcoxon algorithm was used to analyze the marker genes of all clusters, and Group One vs. Rest was used to score them. Specific highly expressed genes with a cluster Log2 Fold Change (log2 FC) > 0.25 and expression in at least 20% of cells were selected as marker genes. Finally, the top genes in the Cell Marker and Panglao DB databases were searched to annotate the cell types of clusters (Supplementary Table S1).

### 2.9 Differentially expressed genes analysis

DEGs were analyzed by using the FindMarkers function in the Seurat package (version 4.0.2). The MAST algorithm was used to calculate statistical significance and controlled False-Discovery Rates (FDRs) using the Benjamini–Hochberg procedure. Then, we set a threshold *p* < 0.05 to filter DEGs and obtained PD up-and down-regulated genes compared to the control for each cluster. These DEGs were into Excel table, and the unique function was used to remove duplicate genes and obtain specific differential genes (Supplementary Table S3).

### 2.10 Functional enrichment analysis

The Gene Ontology Analysis (GO) category database and the Kyoto Encyclopedia of Genes and Genomes (KEGG) database were used for the functional annotation of specific DEGs. The enrichment analysis of GO categories was performed by using the R clusterProfiler (version 3.14.3) package, and the enrichment analysis of the pathways was performed on hypergeometric distribution by using the R phyper function.

### 2.11 Gene Set enrichment analysis

Gene Set Enrichment Analysis (GSEA) was performed using the fgsea package (version 1.23.1) using the hallmark gene set list and KEGG gene set list from MSigDB. For each cluster, genes were ranked by Log2 FC after MAST analysis, and the analysis was performed using the fgsea multilevel command with default settings and seed set at 1000. Gene sets were enriched if the adjusted *p*-value <0.1 (Supplementary Table S2).

### 2.12 PPI network analysis

The STRING database was used for DEG-associated protein interaction analysis and production of Protein–Protein Interaction (PPI) networks. Cytoscape was used to construct the cell differential expression network.

### 2.13 Trajectory analysis using Monocle2

Pseudotime analysis, also called cell trajectory analysis, is commonly used to predict the evolutionary trajectory of cell subtypes and apoptosis pathways or to infer the differentiation trajectory of stem cells during disease progression. In the current study, we used Monocle 2 (version 2.5.4) to order single cells in pseudotime and placed them along an inferred trajectory. In the trajectory analysis, we used genes meeting the thresholds of mean_expression ≥0.08 and dispersion_empirical ≥1 ^*^ dispersion_fit identified by Monocle2 to sort cells in pseudotime order. The reduceDimension () function using the parameters reduction_method = DDRTree and max_components = 2 was applied to reduce dimensions, and the visualization functions plot_cell_trajectory was used to plot the minimum spanning tree on cells. Genes that changed along with pseudotime were calculated (*q*-val < 10^−5^) by the differentialGeneTest function and visualized with plot_pseudotime_heatmap, and the genes were clustered into subgroups according to the gene expression patterns.

### 2.14 Cell–cell communication analysis

To further investigate the intercellular communication changes induced by MPTP, we used R software CellChat (version 1.4.0) to calculate communication networks between subclusters of microglia and other cells. We used the CellChatDB mouse database to predict the communication network, including signaling pathway and ligand–receptor (L-R) pair information, in MPTP and control samples separately and then compared the network differences between these conditions. The interaction number and strength are two key factors, so we used the compareInteractions function to obtain the whole network interaction number and strength differences. Using the aggregateNet function in CellChat, the aggregated cell–cell communication network was calculated, and the signaling from each cell group was visualized. Then, for the conserved signaling pathways, we ranked these pathways according to their Euclidean distance in the shared two-dimensional space. The top pathways indicated more differences between PD and CN. We also compared each signaling pathway’s information flow, which is the sum of communication probability among all cell pairs, to identify different pathway states, including turn off/on, decrease and increase, in one condition compared to the other. Finally, we zoomed in to the L-R pair level and calculated dysfunctional L-R pairs by using differential expression analysis with identifyOverExpressedGenes and netMappingDEG functions. Upregulated and downregulated L-R pairs were detected. All plot functions are from the CellChat package.

## 3 Results

### 3.1 Single-nucleus transcriptome profiling to identify cell populations

**Figure 1:**
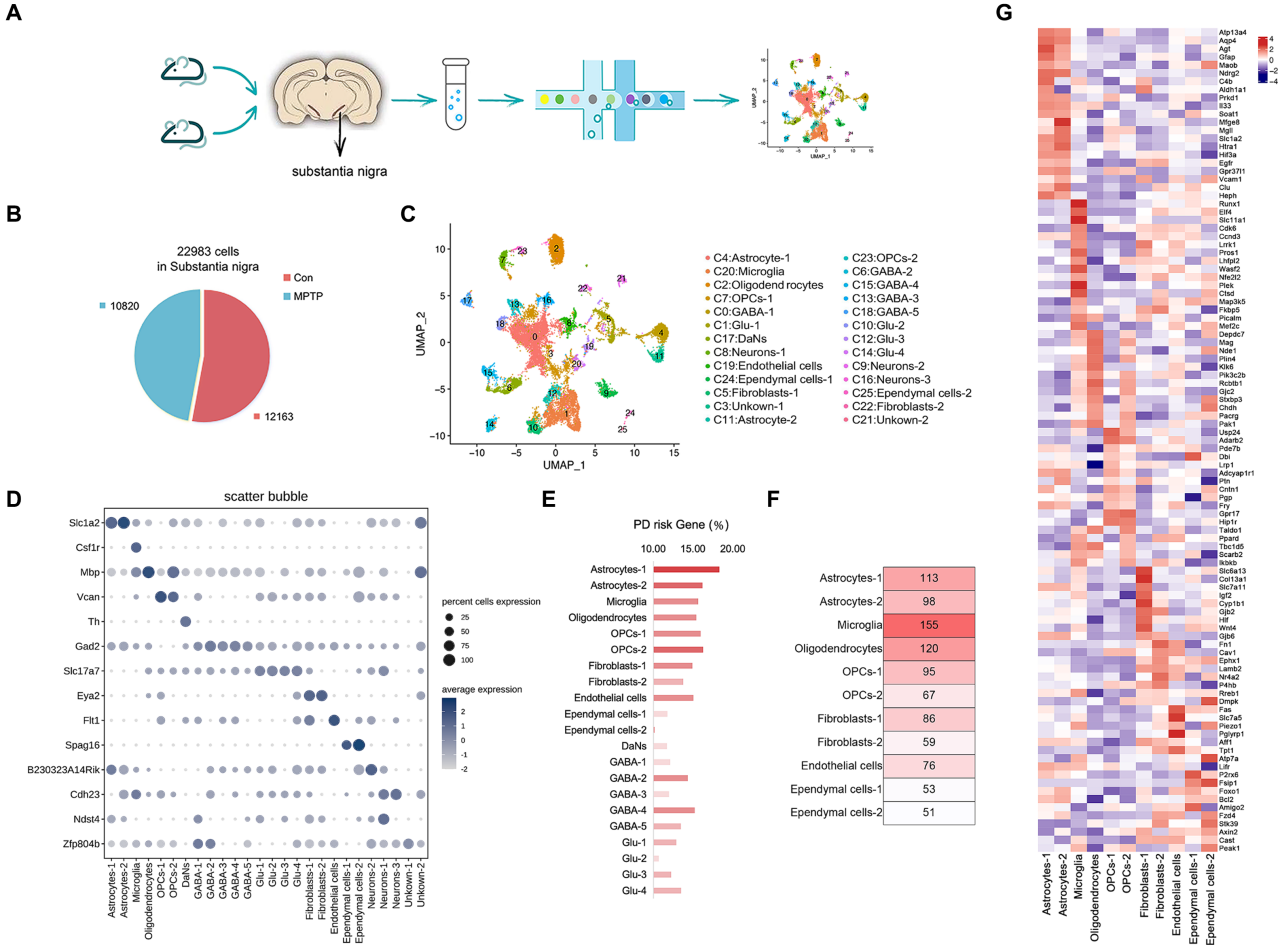
Cell type composition of the mouse SN. **(A)** The experimental approach to SN tissue processing and nuclei extraction. Nuclei suspensions were processed with the 10× Genomics platform and sequenced with an Illumina sequencer. **(B)** The number of high-quality nuclei per sample. Overall, the sample consists of 10,820 nuclei from MPTP and 12,163 nuclei from controls. **(C)** UMAP embedding of the 22,983 mouse SN nuclei; colored by cluster. **(D)** Cell representative marker genes. Expression level (dot size) of marker genes across clusters and the percentage of cell expression (color scale). **(E)** The proportion of PD risk genes in each cluster. **(F)** The number of PD risk genes in non-neuronal cells. **(G)** Heat map of the top 10 % of PD risk genes highly expressed in glial cell types.

PD model mice were generated by intraperitoneal injection of MPTP, and behavioral experiments showed that the motor function of the mice were significantly decreased after injection of MPTP (Supplementary Figure S1). Subsequently, SN samples were isolated from MPTP mice and Con mice to perform snRNA-seq (Figure 1A). We sampled 27,227 cells after filtering out poorly sequenced nuclei and potential doublets and finally obtained 22,983 high-quality nuclei with ~4,300 UMIs and ~ 1,600 genes per nucleus (Figure 1B and Supplementary Figure S2). The UMAP method was used to visually classify all the cells, and 25 clusters were identified (Figure 1C). We artificially annotated most clusters by combining known marker genes and highly expressed genes in each cluster (Supplementary Table S1). Only the top 3 highly expressed marker genes in each cluster were shown in Supplementary Figure S3A. We found that the SN of the mouse comprised 11 major cell types (Figure 1D and Supplementary Figure S3B).

The dataset allowed us to identify the most specific markers for each cluster, many of which are known to play a functional role in these cells. We identified four types of glial cells. Oligodendrocyte (Cluster 2) is characterized by the expression of *Myelin Basic Protein (Mbp)*, encoding a protein needed for proper formation of myelin (Kaushansky et al., 2010) (Supplementary Figure S3C). Oligodendrocyte precursor cells (OPCs) (Clusters 7, 23) highly express *Versican (VCAN)*, encoding chondroitin sulfate proteoglycan, which are predominantly localized in myelinated fiber tracts (Ghorbani et al., 2022) (Supplementary Figure S3D). Astrocytes characteristically express *Solute Carrier Family 1 Member 2 (Slc1a2)* (Clusters 4, 11), encoding the glutamate transporter-1 (GLT-1), which prevents excitatory toxicity by clearing extracellular accumulated glutamate (Qu et al., 2022) (Supplementary Figure S3E). Microglia (Cluster 20) is characterized by the expression of *Colony Stimulating Factor 1 Receptor (Csf1r)*, a receptor kinase essential for microglia survival and proliferation (Johnson et al., 2023) (Supplementary Figure S3F). Regarding neuronal cells, we identified four cell types: glutamatergic neurons (Glu) (Clusters 1, 10, 12, 14) with highly express *Solute Carrier Family 17 Member 7 (Slc17a7)*, encoding sodium-dependent phosphate transporters, which play an important role in glutamate delivery in neurons (Du et al., 2020) (Supplementary Figure S3G). GABAergic neurons (GABA) (Clusters 0, 6, 15, 13, and 18) were characterized by the expression of *Glutamate Decarboxylase 2 (Gad2)*, which encodes glutamate decarboxylase and is widely distributed in the axon terminus of inhibitory neurons (Lee et al., 2019) (Supplementary Figure S3H). The expression of *Tyrosine Hydroxylase (TH)* was characteristic of DaNs (Cluster 17), which encode rate-limiting enzymes in catecholamine synthesis and are involved in the conversion of tyrosine to dopamine (Garritsen et al., 2023) (Supplementary Figure S3I). Some were uncommon, such as *N-deacetylase and N-sulfotransferase 4 (Ndst4)* (markers of deep neurons), which was highly expressed in Cluster 9 (Cid et al., 2021); *B230323A14Rik* (a long noncoding RNA in neurons), which was highly expressed in Cluster 8 (Hajdarovic et al., 2022); and *Cadherin-23* (*Cdh23*) (involved in the differentiation of neurons), which was highly expressed in Cluster 16 (Lord and Cruchaga, 2014) (Supplementary Figures 3J–L). Less abundant cell types were also observed, including Endothelial cells (*Fms Related Receptor Tyrosine Kinase 1*, *Flt1*), Ependymal cells (*Sperm Associated Antigen 16, Spag16*), and Fibroblasts (*EYA Transcriptional Coactivator and Phosphatase 2, Eya2*) (Supplementary Figures S3M–O).

### 3.2 Exploration of MPTP-associated cells

We compared the proportions of cell types between MPTP and Con groups. The proportions of Oligodendrocytes, Glu-3 and Ependymal cells-1 were significantly reduced in the MPTP group. Astrocytes, GABA-2, GABA-4, Glu-2, Glu-4, Fibroblasts and Ependymal cells-2 were increased in the MPTP group. (Supplementary Figures S4A,B). DaNs comprised only 426 cells (Supplementary Figure S4B), this limiting the comparability between MPTP and Con groups. However, the general marker genes of DaNs all showed high expression in our data (Supplementary Figure S4C). DaNs are defined by the expression of general markers, such as TH and Dopa Decarboxylase (Ddc), which are necessary for the stepwise production of Dopamine from its precursor L-Tyrosine (Björklund and Dunnett, 2007). In addition, some other informative DaNs markers were also highly expressed, such as Dopamine Transporter (Dat; Slc6a3) (Lammel et al., 2015), DaNs development related gene Nuclear Receptor Subfamily 4 Group A Member 2 (Nr4a2) (Andersson et al., 2007), Engrailed 1 (En1) and En1 downstream target gene Pituitary Homeobox 3 (Pitx3) (Veenvliet et al., 2013), which are essential for development of DaNs as well as for the synthesis and handling dopamine in differentiated neurons.

In addition, we performed quantitative immunofluorescence imaging analysis of the DaNs marker TH in MPTP and Con tissues. The result showed that the number of TH-positive cells in the SN of MPTP was significantly reduced compared to the Con group (Supplementary Figure S4G). DaNs are mainly located in the SN and Ventral Tegmental Area (VTA) (Mesman and Smidt, 2020). Umap and heat maps showed that Aldehyde Dehydrogenase 1 Family Member A1 (Aldh1a1), SRY-box Transcription Factor 6 (Sox6), Neuron-derived Neurotrophic Factor (Ndnf), and Anti-inflammatory Factors Annexin A1 (Anxa1) were highly expressed in DaNs (Supplementary Figures S4D,F), which have been reported to be found in DaNs of the ventral SN (Poulin et al., 2020). Sox6 is an important transcription factor involved in the differentiation and development of DaNs (La Manno et al., 2016). Aldh1a1 mediates the oxidation of reactive Dopamine-3,4-dihydroxyphenylacetaldehyde (Dopal) into 3,4-dihydroxyphenylacetic Acid (Dopac), which is then degraded to form homovanillic acid in DaNs (Liu et al., 2014). DaNs located in the VTA are expressing Vesicular GABA Transporter (Vgat; Slc32a1), Vesicular Glutamate Transporter 2 (Vglut2; Slc17a6), and Orthodenticle Homeo-box 2 (Otx2) (Poulin et al., 2020). Slc32a1, Slc17a6, and Otx2 were less expressed in SN (Supplementary Figure S4E). This suggested that DaNs were derived from SN in our data.

GSEA of cell type marker genes demonstrated functional characteristics of the corresponding cell type. GSEA analysis demonstrated that DaNs was most closely related to PD (Supplementary Figure S5 and Supplementary Table S2). And GO enrichment analysis of differential genes in DaNs showed that “mitochondrial function” and “kinase activity” were significantly enriched (Supplementary Figure S4H). MPP is released from glial cells and taken up by DaNs through the dopamine transporter. MPP accumulates in mitochondria leads to kinase activation, ATP depletion and Reactive Oxygen Species (Ros) production (Hu and Wang, 2016; Dionísio et al., 2021). This was consistent with the results of GO enrichment analysis.

We next sought to locate the cell type that significantly contributes to PD progression. To achieve this, we used the DisGeNET database^1^ to assess whether the expression patterns of PD risk genes are cell specific (Piñero et al., 2017). The platform is aggregated from multiple sources, including curated repositories, Genome Wide Association Studies (GWAS) catalogs, animal models, and the scientific literature. We assessed the percentage of PD risk genes in DEGs of each cluster. The results showed that PD risk genes were significantly enriched in non-neuronal cells (Figure 1E), and microglia had the highest number of PD risk genes in the MPTP (Figure 1F and Supplementary Table S2). In addition, we provide the top 10 % highly expressed PD risk genes in glial cell types (Figure 1G). Therefore, we speculate that microglia are closely related to the occurrence and development of PD, which will be the focus of further research.

### 3.3 Microglia-specific DEGs and functional analysis

**Figure 2:**
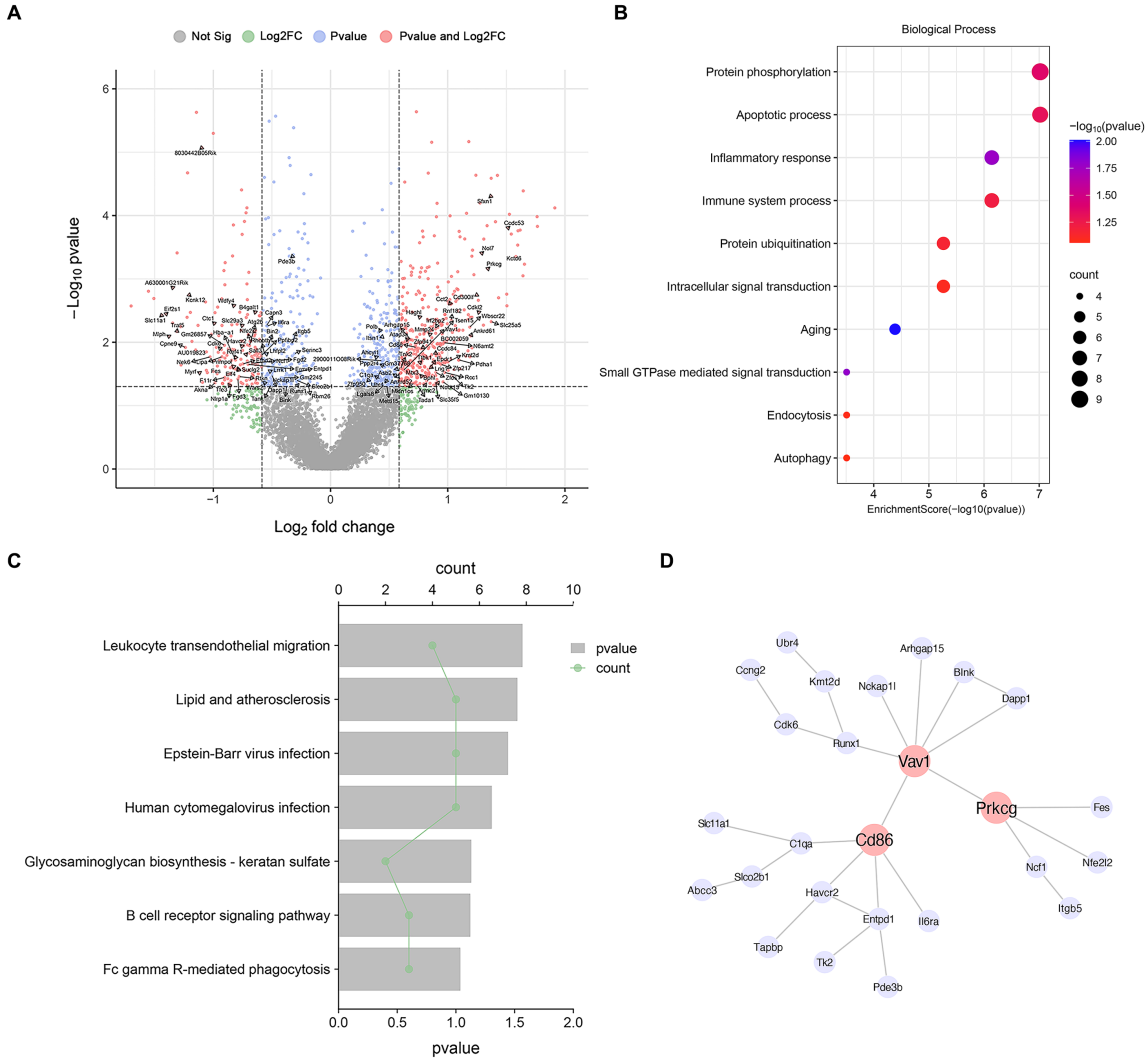
Microglia-specific DEG analysis. **(A)** Volcano map of DEGs in microglia. The annotated genes are microglia-specific DEGs. **(B)** GO terms associated with microglia-specific DEGs. **(C)** KEGG terms associated with microglia-specific DEGs. **(D)** Microglia-specific DEGs PPI network analysis diagram.

Many cell types can share common DEGs in MPTP compared with Con, but their fold change in different cell types were different. So, searching specific DEGs plays an important role in identifying cell functions. We defined genes with |logFC| > 0 and *p* < 0.05 only in microglia as microglia-specific DEGs. After screening, 126 microglia-specific DEGs were obtained and marked in the volcano diagram. (Figure 2A and Supplementary Table S3). To gain insight into the underlying biological processes of DEGs, we analyzed transcript lists of microglia-specific DEGs using GO functional annotation. These genes are mainly involved in “Inflammatory response” (*Polb, Nlrp1a, Ncf1, Slc11a1, Lipa, Havcr2, Nfe2l2*), “Immune system processes” (*Cd86, C1qa, Nlrp1a, Tfe3, Cd300lf, Serinc3, Havcr2*), “Aging” (*Ctc1, Polb, C1qa, Eif2s1, Nfe2l2*), and “Autophagy” (*Lgals8, Wdfy4, Rnf41, Atg2b*) (Figure 2B). Then, KEGG pathway analysis revealed that these genes were enriched in “lipid and atherosclerosis” (*Ncf1, Eif2s1, Tank, Vav1, Nfe2l2*), “B-cell receptor signaling pathway” (*Blnk, Dapp1, Vav1*), and “Fc gamma R-mediated phagocytosis” (*Prkcg, Ncf1, Vav1*) (Figure 2C and Supplementary Table S3).

Then, we constructed cell type-specific networks by using PPI analysis and identified several highly interconnected genes. We constructed a PPI network of microglia-specific genes using the STRING database and Cytoscape software. We found that Cd86, Vav1, and Prkcg are at the hub position and are most widely associated with other genes and play an important role in regulating the function of microglia (Figure 2D). CD86 is associated with microglial inflammation (Zhang et al., 2023). Vav1 is a guanine nucleotide exchange factor associated with microglial activation, plays a key role in cell signaling, and plays an important role in inflammation in microglia (Li et al., 2023). Protein kinase C gamma (Prkcg) belongs to the PKC serine-and threonine-specific protein kinase family and is involved in a variety of signaling pathways. It is widely expressed in many cell types, including microglia (Wadsworth and Goldfine, 2002). PKC activation is an important mediator of microglial activation, and by inhibiting PKC signaling, microglial activation is inhibited (Alawieyah Syed Mortadza et al., 2018).

**Figure 3:**
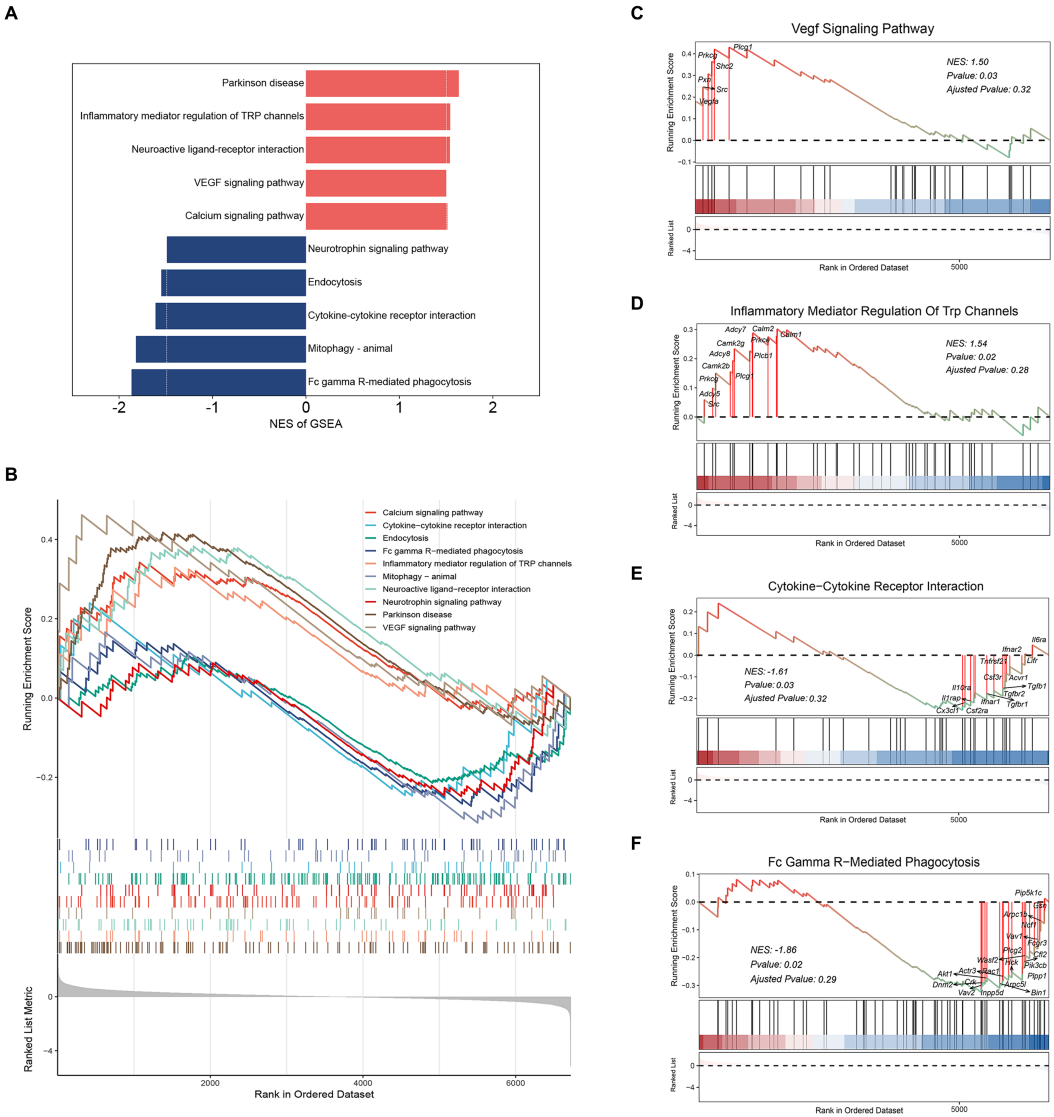
GSEA analysis for microglia DEGs. **(A)** GSEA two-way bar plot microglia DEGs. **(B)** Visualization of GSEA results for microglia DEGs. **(C)** Visualization of GSEA results for “VEGF signaling pathway.” **(D)** Visualization of GSEA results for “Inflammatory mediator regulation of TRP channels.” **(E)** Visualization of GSEA results for “Cytokine-cytokine receptor interaction.” **(F)** Visualization of GSEA results for “Fc gamma R-mediated phagocytosis.” Demonstrating the first 5 pathways, NES > 0, activated; NES < 0, inhibited.

GSEA using DEGs were applied to further investigate cellular processes in microglia. We analyzed the Top 10 pathways with the greatest enrichment scores (Figures 3A,B). We observed that “Parkinson’s disease,” “Inflammatory mediator regulation of TRP channels,” “Neuroactive ligand-receptor interaction,” “VEGF signaling pathway” and “Calcium signaling pathway” exhibited positive Normalized Enrichment Scores (NES) (Figures 3C,D and Supplementary Figures S6A–C). In contrast, notable negative NES were enriched in “Fc gamma R-mediated phagocytosis,” “Mitophagy-animal,” “Cytokine-cytokine receptor interaction,” “Endocytosis” and “Neurotrophin signaling pathway” (Figures 3E,F and Supplementary Figures S6D–F). These results indicated that Transient Receptor Potential (TRP) and Vascular Endothelial-derived Growth Factor (VEGF) inflammation-related pathways were activated in microglia (Figures 3C,D). At the same time, phagocytosis and cytokine interaction were inhibited (Figures 3E,F), which was consistent with the previous GO and KEGG analysis results. In conclusion, these data will deepen our understanding of the regulatory basis of microglia function.

### 3.4 Analysis of cell–cell communication in MPTP microglia

**Figure 4:**
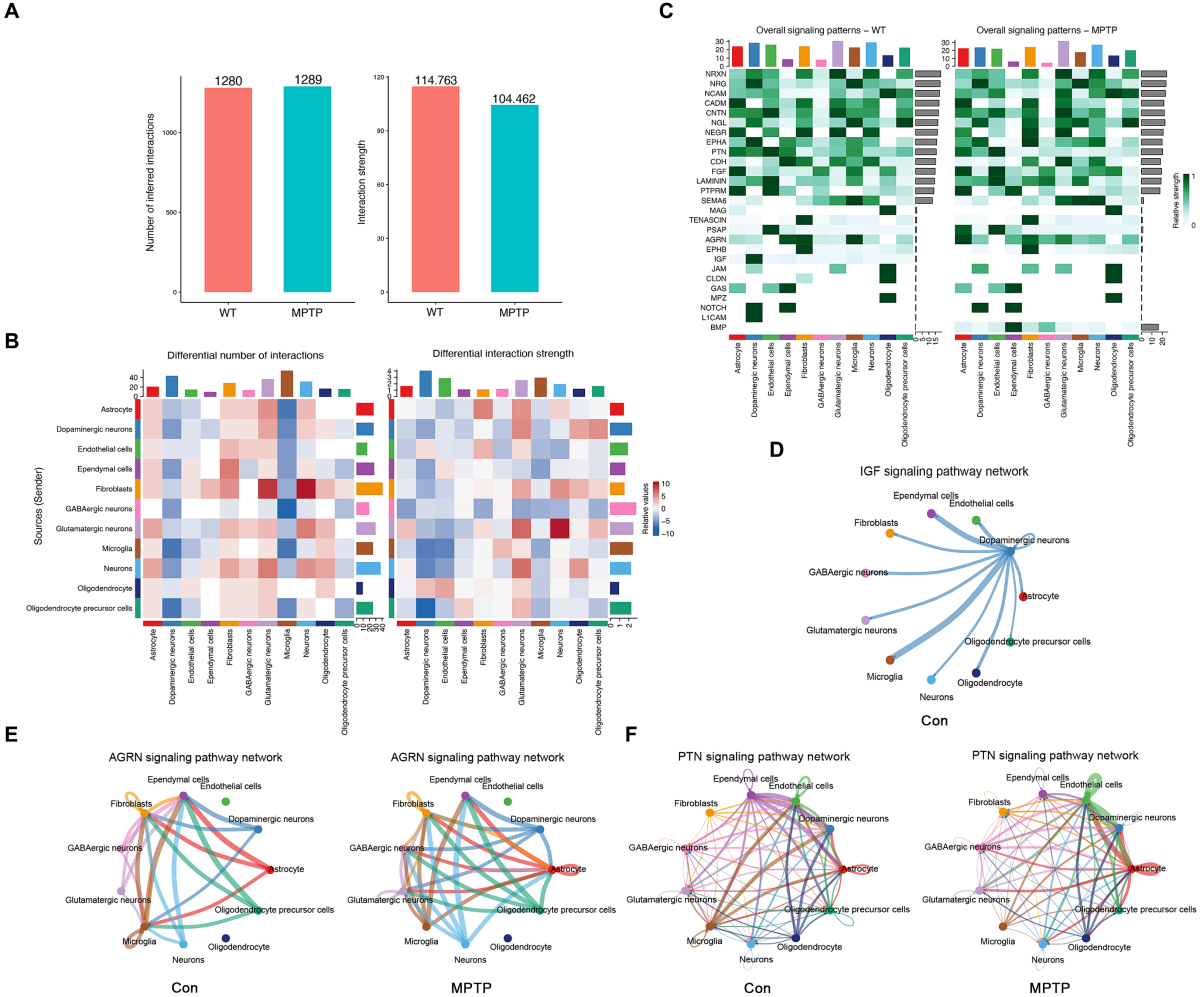
Characterization of cell communications among microglia. **(A)** The number and strength of intercellular ligand–receptor interactions in the MPTP and Con. **(B)** Heatmaps of the interaction numbers (left) and strengths (right) in the MPTP and Con groups. **(C)** Heatmaps of the overall signaling pathway for each cell in Con (left) and MPTP (right). **(D)** Circle plots show the IGF signaling pathway among microglia and other cells in the Con group. **(E)** Circle plots show the AGRN signaling pathway among microglia and other cells in the Con (left) and MPTP (right) groups. **(F)** Circle plots show the PTN signaling pathway among microglia and other cells in the Con (left) and MPTP (right) groups.

Integrating KEGG pathway analysis and GSEA enrichment analysis of microglia suggests that MPTP mice may have changes in intercellular communication, including “cytokine–cytokine receptor interactions” and “B-cell receptor signaling pathways.” To further explore the interactions between microglia and other cells, we used CellChat to infer the communication networks between cells. We found that the number of inferred interactions in the MPTP group did not change significantly, while the interaction strength was weaker in the MPTP group than in the Con group (Figure 4A). Moreover, the number and intensity of communication between microglia and other cells were most significantly reduced (Figure 4B). Next, we wondered which signaling pathways and L-R pairs altered the microglial communication network. We found that some pathways, such as the Insulin-like Growth Factor (IGF) pathway, were turned off in the MPTP. Some signaling pathways, such as the Agrin (AGRN) and Pleiotrophin (PTN) pathways, were decreased in the MPTP (Figure 4C). Moreover, we studied the detailed changes in the outgoing and incoming signaling across all pathways using pattern recognition analysis.

The IGF signaling pathway was abolished in the MPTP group (Figure 4C). As shown in Figure 4C, ligand Igf1 is released by DaNs, and microglia received more than other cells. (Figure 4D and Supplementary Figures S7A,B). We also discovered AGRN and PTN, whose signals are also greatly altered in the MPTP (Figure 4C). AGRN signaling consists of a ligand AGRN and receptor Dystroglycan 1 (Dag1). As shown in Figure 4E, under physiological conditions, microglia release and receive Agrn from other cells. In the MPTP, microglia only release Agrn and do not accept Agrn signaling from other cells (Figure 4E and Supplementary Figures S7C,D). In the PTN signal, protein tyrosine phosphatase receptor type Z1 (Ptprz1) is the main receptor. In MPTP, the PTN signaling of microglia was reduced, and the signals received from other cells disappeandared (Figure 4F and Supplementary Figures S7E,F). Our analysis suggests that alterations in IGF, AGRN, and PTN signaling in microglia may be an important factor leading to changes in microglial function. Alterations in intercellular communication may provide a theoretical basis for further exploration of the pathogenesis of PD.

### 3.5 Subpopulation reconstruction and trajectory analysis of microglia

**Figure 5:**
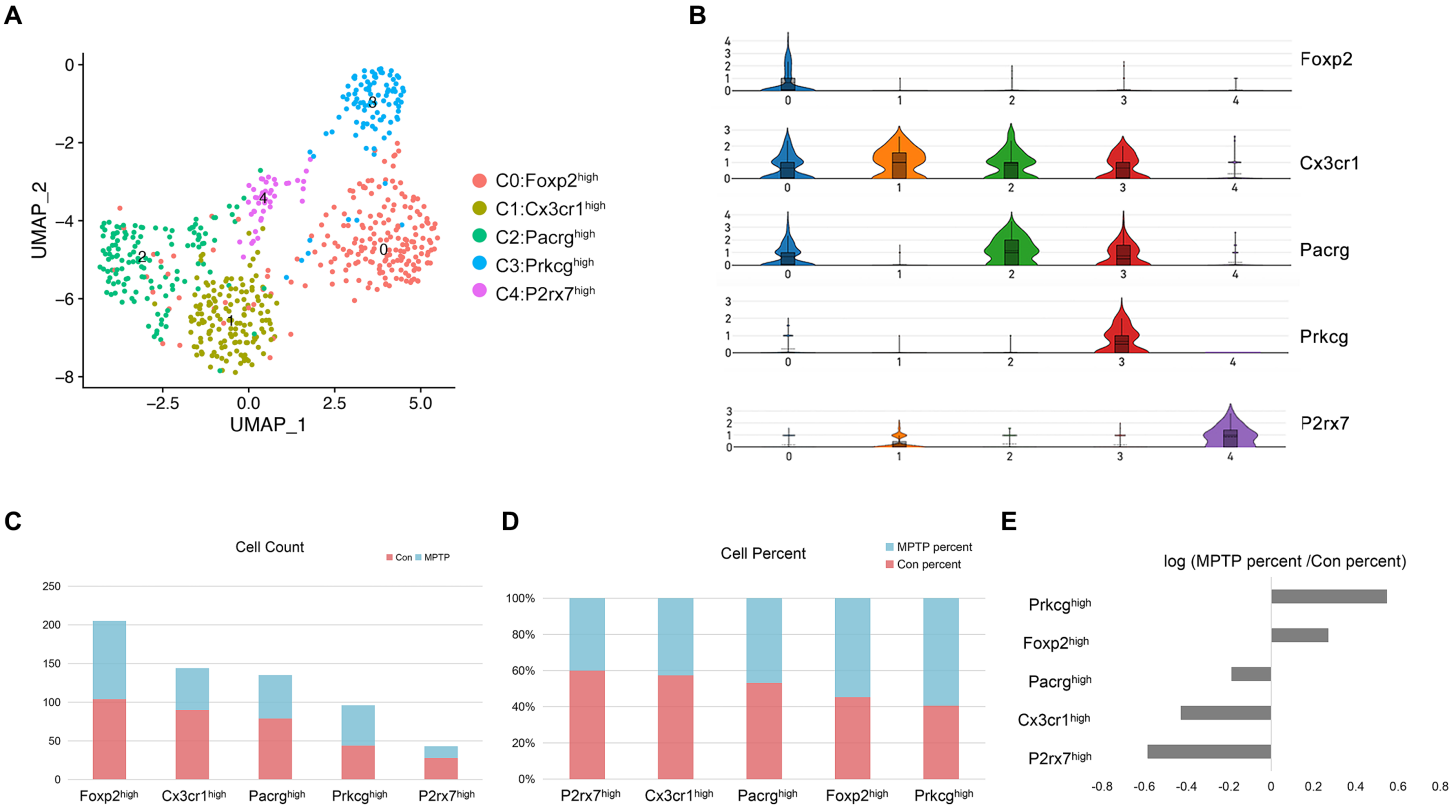
Microglia subpopulation reconstruction. **(A)** UMAP embedding of the microglia nuclei; colored by cluster. **(B)** Violin plot of the relative expression of marker genes in microglial clusters. **(C)** The number of profiled nuclei per cluster. **(D)** The proportion of MPTP-and control-profiled cells per cluster. **(E)** Log ratio of the average fraction in the MPTP group vs. the Con.

To gain a deeper understanding of the heterogeneity of microglia cluster, we reclustered the subset of microglia in Con and MPTP. The results showed that microglia could be reclustered into 5 clusters based on marker genes identified by the R package Seurat. These microglial subpopulations characterized by high expression of Forkhead Box P2 (Foxp2), C-X3-C Motif Chemokine Receptor 1 (Cx3cr1), Parkin Co-regulated Gene (Pacrg), Prkcg, and P2X purinoceptor 7 (P2rx7) (Figures 5A,B and Supplementary Table S4). Of these, Cluster 0 was the most abundant and had high expression of Foxp2 (Figure 5C). Recent studies have shown that Foxp2 is expressed in microglia (Ma et al., 2022). Cluster 1 expressed most genes associated with microglial homeostasis, such as Cx3cr1, P2ry13, and P2ry12, but it did not have specific markers compared to other subpopulations (Masuda et al., 2020). Cluster 2 highly expresses Pacrg, which encodes the Parkin coregulated protein, a protein found in Lewy bodies and related to aggresome formation and increased autophagy (Taylor et al., 2012). The characteristic gene of Cluster 3 was Prkcg, and the cell proportion in the MPTP group was significantly higher than that in the control group (Figures 5D,E). As mentioned earlier, Prkcg is an important PD risk gene in microglia, and PKC has been reported to promote increased microglial neuroinflammation (Liu et al., 2019). Cluster 4 specifically expressed P2rx7, and it was significantly reduced in MPTP mice. P2rx7 is an ATP-evoked Na^+^/Ca^2+^ channel predominantly expressed in microglia (Ruan et al., 2020). It can modulation of microglia death and cytokine release (He et al., 2017).

**Figure 6:**
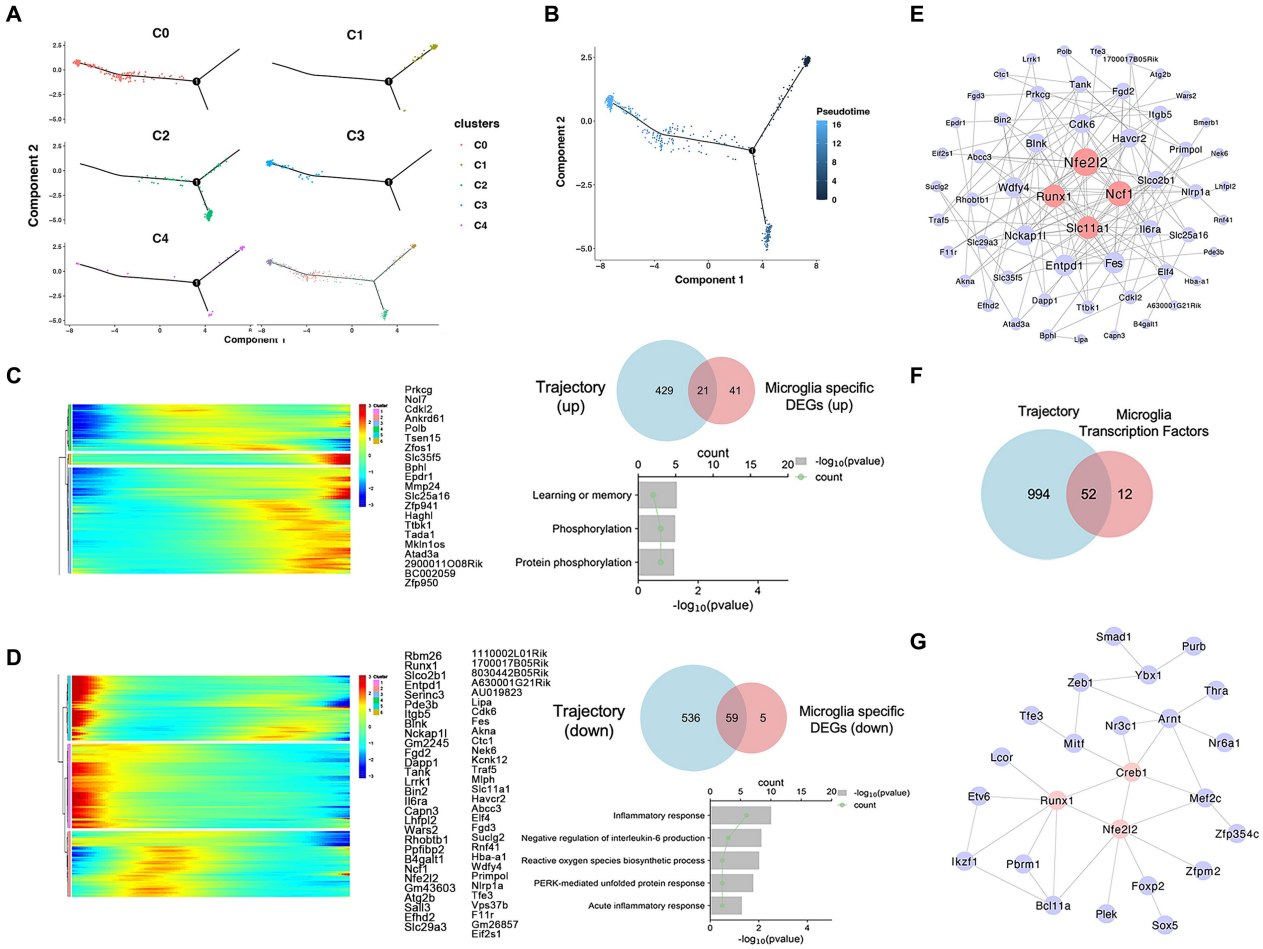
Trajectory analysis of microglia. **(A)** Monocle2 pseudotime analysis was performed, and the position of each subtype on the pseudotime trajectory and each subtype are marked with a different color. **(B)** The metastatic trajectory of distinct cell clusters is shown. The color gradient indicates pseudotime progression. **(C)** The intersection of microglia-specific upregulated DEGs and trajectory-associated genes. Additionally, the GO molecular enrichment of the intersected genes is presented. **(D)** The intersection of microglia-specific downregulated DEGs and trajectory-associated genes. Additionally, the GO molecular enrichment of the intersected genes is presented. **(E)** 80 specific DEGs associated with activation trajectories in microglia PPI network analysis diagram. **(F)** The intersection of differentially expressed transcription factors and trajectory-associated genes in microglia. **(G)** 52 transcription factors associated with activation trajectories in microglia PPI network analysis diagram.

To investigate changes in the state of microglia in MPTP mice, we reconstructed their activation trajectories. Five subpopulations fit into a continuum in the UMAP projection, and we used the Monocle2 method to reconstruct the cell trajectory structure comprising these major subpopulations (Figure 6A). Activation of Prkcg can lead to inflammation in microglia, and the increased proportion of Prkcg^high^ microglia coincided with the reduced proportion of homeostatic microglia (Figure 5E). From this, we estimated that the activation trajectory of microglia spans from Cx3cr1^high^ cells (Cluster 1) toward Prkcg^high^ cells (Cluster 3) (Figure 6B). The trajectory accurately captures the transition of microglia from homeostasis to an active state. To further characterize the molecular phenotype of this activated microglial state, we identified specific DEGs associated with activation trajectories in microglia and functionally enriched them in GO enrichment analysis. An overlay of microglia-specific DEGs and of such genes defining the microglia trajectory identified 21 upregulated and 59 downregulated genes across the trajectory (Figures 6C,D). The upregulated genes were associated with “protein phosphorylation,” while the downregulated genes were associated with “inflammatory responses” (Figures 6C,D and Supplementary Table S5). Among these genes, Nuclear Factor (erythroid-derived 2)-Like 2 (Nfe2l2), Neutrophil Cytosolic Factor 1 (Ncf1), Solute Carrier Family 11 Member 1 (Slc11a1), and Runt-related Transcription Factor 1 (Runx1) were most widely associated with other genes (Figure 6E). Cell type diversity is achieved through the expression of transcriptional regulators that regulate cell status. Therefore, we further investigated the effects of transcription factors on microglial activation from the perspective of transcriptional regulation. We identified a total of 52 transcription factors that vary with microglial activation trajectory (Figure 6F). We constructed PPI network for these transcription factors, the results show that Nfe2l2, Creb1, and Runx1 were at the hub position (Figure 6G). Therefore, we hypothesized that Nfe2l2 and Runx1 might regulate microglial activation in MPTP.

## 4 Discussion

Emerging scRNA-seq approaches have become instrumental in deciphering the intricate heterogeneity and composition of cell types of complex human diseases including PD (Ma and Lim, 2021). In this study, we profiled a large number of nuclei in the SN from both MPTP and Con mice, aiming to characterize the cell-and disease-specific molecular features associated with PD in the whole SN. Our data reveal cell type-specific molecular alterations and disruption of cell–cell communication networks in MPTP-induced model mice. Our results highlight the cell heterogeneity and molecular basis for the complexity underlying disease mechanism.

When assessing DaNs in our snRNA-seq data, we did not observe a significant loss in MPTP group. The low abundance of DaNs may hampered this comparison in the SN, and this may be due to the technical limitations. We obtained the SN at a coronal section 3–4 mm posterior to the anterior fontanelle (behind the hippocampal region), about 2–3 mm thick. The color of the SN is different from other brain regions, and it has a relatively clear boundary. During sampling, we removed a portion of the excess tissue to ensure SN rigor. This may have resulted in some cell loss and low abundance of DaNs in the SN. However, it is necessary to verify the DaNs is originated from SN. First, image analysis of SN sections in immunofluorescence-labeled TH confirmed a significant loss of DaNs. Secondly, in addition to general DaNs marker genes such as TH, Slc6a3, En1, Nr4a2, Pitx3 and Ddc showed high expression in DaNs. SN specific DaNs marker genes including Sox6, Aldh1a1, Ndnf and Anxa1 all showed high expression in DaNs, which clarified the DaNs was origined from SN in our data. Finally, in GSEA and GO enrichment analyses, we also observed that DaNs were closely related to PD, and “mitochondrial function” and “kinase activity” were significantly enriched in DaNs.

In our dataset, PD risk genes were significantly enriched in microglia. Microglia are innate immune cells of the central nervous system that play an important role in mediating inflammation, maintaining brain homeostasis, and clearing cell debris (Colonna and Butovsky, 2017). Under normal circumstances, microglia are in a resting state, play an immune surveillance role, and maintain the normal physiological function of the central nervous system. When microglia are activated after tissue injury or infection, they perform phagocytosis and secretory functions by changing morphological migration to restore tissue homeostasis (El Khoury, 2010). In our results, inflammation-related pathways such as TRP and VEGF were activated and phagocytosis, autophagy, and cytokine interaction were inhibited. MPTP has been shown to cause pooling of blood in the brain microvasculature and decrease the permeability of the blood–brain barrier, and blood–brain barrier dysfunction is involved in the course of PD (Raza et al., 2019). VEGF plays in regulating angiogenesis and can mediates blood–brain barrier disruption in PD (Wood, 2022). In microglia, VEGF can induce NLRP3 inflammasome activation, increase microglial inflammation, and then destroy the integrity of the blood–brain barrier (Ronaldson and Davis, 2020; Marneros, 2023). TRP is a non-selective cation channel that can increase microglial inflammation by increasing intracellular Ca^2+^(Kong et al., 2017; Zhang et al., 2021). In addition, Microglia are brain professional phagocytes mainly finalized to clearance of apoptotic or necrotic cells and removal of unfolded proteins (Janda et al., 2018). It is well known that microglia can delay the progression of PD by engulfing α-synuclein. Microglia can also play a neuroprotective role by clearing α-synuclein and inflammasomes through autophagy (Choi et al., 2022).

Dysregulation of intercellular communication may be a key factor in the alteration of microglia function. We found changes in cell ligand receptor signaling in both KEGG and GSEA enrichment analyses of microglia-specific DEGs. We analyzed the connections between microglia and other cells via CellChat. We found that many signaling pathways were significantly reduced in microglia, including IGF, AGRN, and PTN. IGF is a neurotrophic protein that regulates brain development, cell survival, and the clearance of agglutinin through autocrine, paracrine, and endocrine mechanisms (Ivan et al., 2023). In the central nervous system, Igf1 is highly expressed in a subset of DaNs (Chung et al., 2005; Poulin et al., 2014). Igf1r is expressed by virtually all resident and invading inflammatory cells, with Igf1 in turn being able to modulate the function of different inflammatory players (Ivan et al., 2023). AGRN is a 210-kDa heparan sulfate proteoglycan that can function as a transmembrane or a secreted protein in the extracellular matrix. Dag1 is a cell adhesion molecule known to be essential for skeletal muscle integrity (Tian et al., 2020). It has also recently been reported that it regulates synaptic plasticity (Xie et al., 2022). PTN is a neurotrophic factor that regulates glial responses in animal models of different types of central nervous system injuries (Herradon et al., 2019). We speculate that changes in microglial communication may be important factors leading to microglial activation, and they may be potential markers of PD pathology.

To investigate the changes in microglia states in MPTP-induced mice, we subclustered these cells and reconstructed their activation trajectories. However, we found that Prkcg^high^ cells (Cluster 3) and some Foxp2^high^ cells (Cluster 0) were located at the end of the track. Foxp2 encodes a key transcription factor that plays a role in the development of circuits related to speech, language, and human brain evolution (Caglayan et al., 2023). Recent studies have shown that the expression of Foxp2 has also been detected in microglia, but its specific function has not been reported (Ma et al., 2022). By inferring the activation trajectory of microglia, we observed an increase in the proportion of microglia from homeostatic to activated states. The increased proportion of Prkcg^high^ microglia coincided with the reduced proportion of homeostatic microglia. There are many studies reported that Prkcg are associated with microglial activation (van der Vorst et al., 2017; Guo et al., 2021b; Kim and Kornberg, 2022). GO analysis of microglia-specific DEGs upregulated and downregulated with trajectory identified terms such as “inflammatory response” and “phosphorylation” in microglia. Then we identified the transcription factors most closely associated with microglial activation: Nfe2l2 and Runx1. Nfe2l2 is a major transcription factor that regulates the antioxidant response. Upon translocation into the nucleus, Nfe2l2 binds to genes containing regulatory antioxidant response element sequences to enhance the transcription of a subset of genes involved in detoxification and antioxidant responses (Vilhardt et al., 2017). In microglia, Nfe2l2-deficiency promotes inflammatory factors expression whilst inhibiting anti-inflammatory factors expression in response to MPTP (Rojo et al., 2010; Simpson and Oliver, 2020). Runx1 is an indispensable regulator of the differentiation process during embryonic development (Ginhoux et al., 2010). However, it has been reported that Runx1 is not only a regulator of differentiation, but also regulates the proliferation and homeostasis of postnatal microglia (Zusso et al., 2012). Runx1 might play an important function in microglia by modulating the transition of amoeboid microglia to ramified ones (Zusso et al., 2012). Therefore, Runx1 is a non-redundant transcription factor that is important for the activation and resting states of microglia. Runx1 knockdown by small interfering RNA in BV-2 cells strongly promoted microglial inflammation (Zusso et al., 2012). However, the function of Runx1 in PD is still unclear and needs further study.

In conclusion, our study reinforces the relevance of microglial inflammation and PD. Initially, we found significant specific DEGs and PD risk genes enrichment in microglia. In addition, the cell–cell communication relationship between microglia and other cells was investigated in detail, and PD-related signaling factors and L-R pairs were identified. Finally, trajectory analyses in the microglial populations identified that inflammatory responses are activated with trajectory, and Nfe2l2 and Runx1 may be the major triggers of inflammatory signaling in PD. Taken together, our work at least partially supports the changes in microglial state and genes in the SN of MPTP model mice, and we hope that this study can provide references for the pathogenesis of PD.

## Data availability statement

The original contributions presented in the study are included in the article/Supplementary Material. Further inquiries can be directed to the corresponding authors.

## Ethics statement

The animal study was approved by Animal Ethics and Welfare Committee of Hebei Medical University (IACUC-Hebmu-P2022153). The study was conducted in accordance with the local legislation and institutional requirements.

## Author contributions

QL: Investigation, Visualization, Writing – original draft. ZL: Investigation, Visualization, Writing – original draft. WX: Formal analysis, Methodology, Software, Writing – original draft. YL: Data curation, Software, Writing – original draft. HW: Formal analysis, Methodology, Writing – original draft. SZ: Funding acquisition, Writing – original draft. WW: Data curation, Writing – original draft. JH: Data curation, Writing – original draft. DG: Project administration, Supervision, Writing – review & editing. JY: Data curation, Funding acquisition, Writing – review & editing. LW: Funding acquisition, Resources, Supervision, Writing – review & editing.

## Footnotes

^1^www.disgenet.org
