# Supplementary material for: Single-cell sequencing of the substantia nigra reveals microglial activation in a model of MPTP
Source: Front Aging Neurosci. 2024 Jun 17;16:1390310. doi: 10.3389/fnagi.2024.1390310 (PMC11215054; doi:10.3389/fnagi.2024.1390310)
Supplement: Supplementary file 6 [file Data_Sheet_1.PDF]

Supplementary Figures

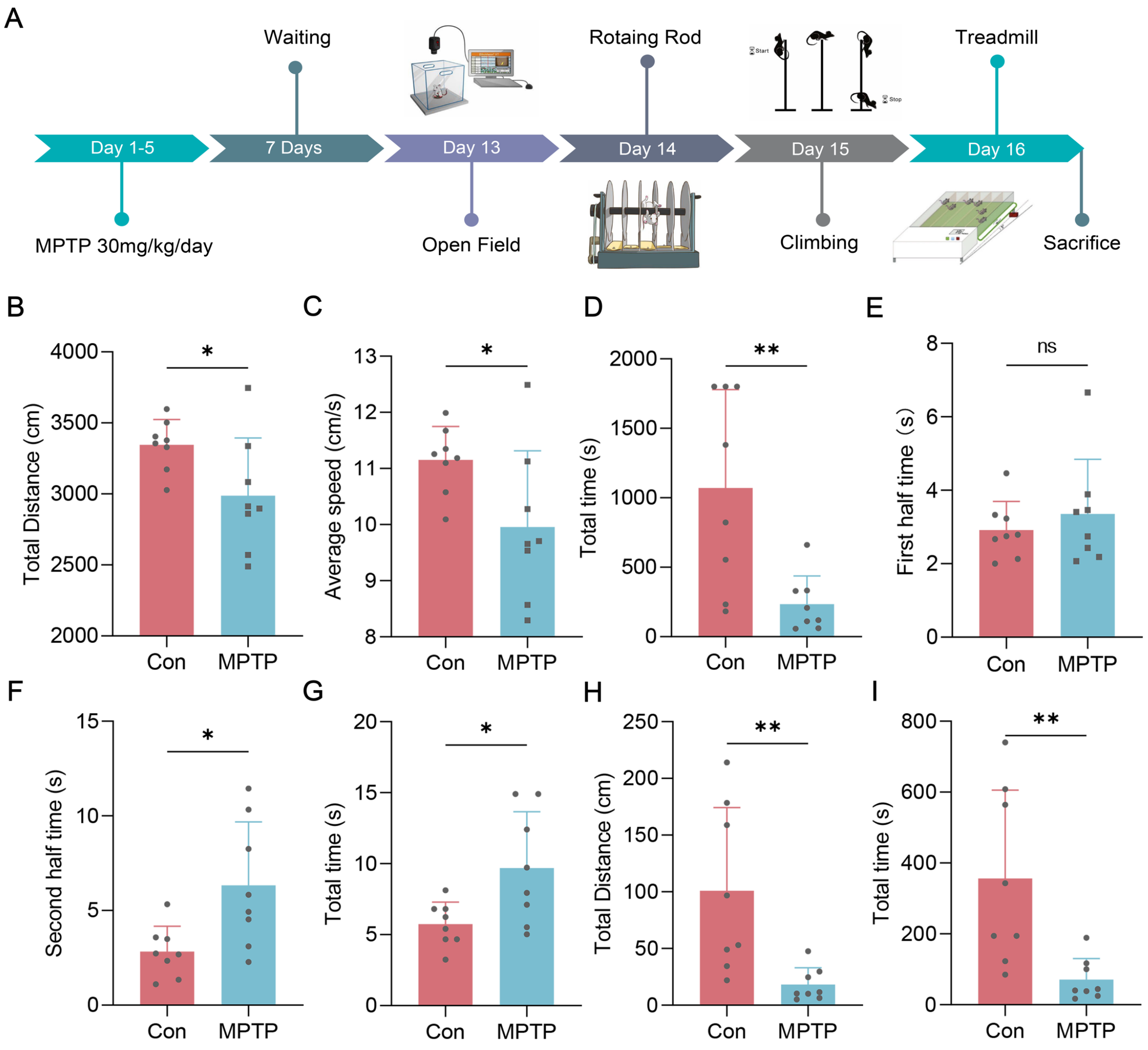

Supplementary Figure 1. Behavioral experiments showed that PD model mice had motor dysfunction. (A) Schematic description of the experimental process. MPTP (30 mg/kg) was injected intraperitoneally for 5 consecutive days, and behavioral experiments were performed 7 days later. **(B-C)** Open field test, total distance traveled, and average speed of traveling (n=4). **(D)** Rotating rod test, the time spent on rod (n=4). **(E-G)** Climbing test, the time spent crawling down (n=4). **(H-I)** Treadmill test, the distance of running, the time of running (n=4). Data are presented as the mean ± SD. $*P < 0.05$, $**P < 0.01$.

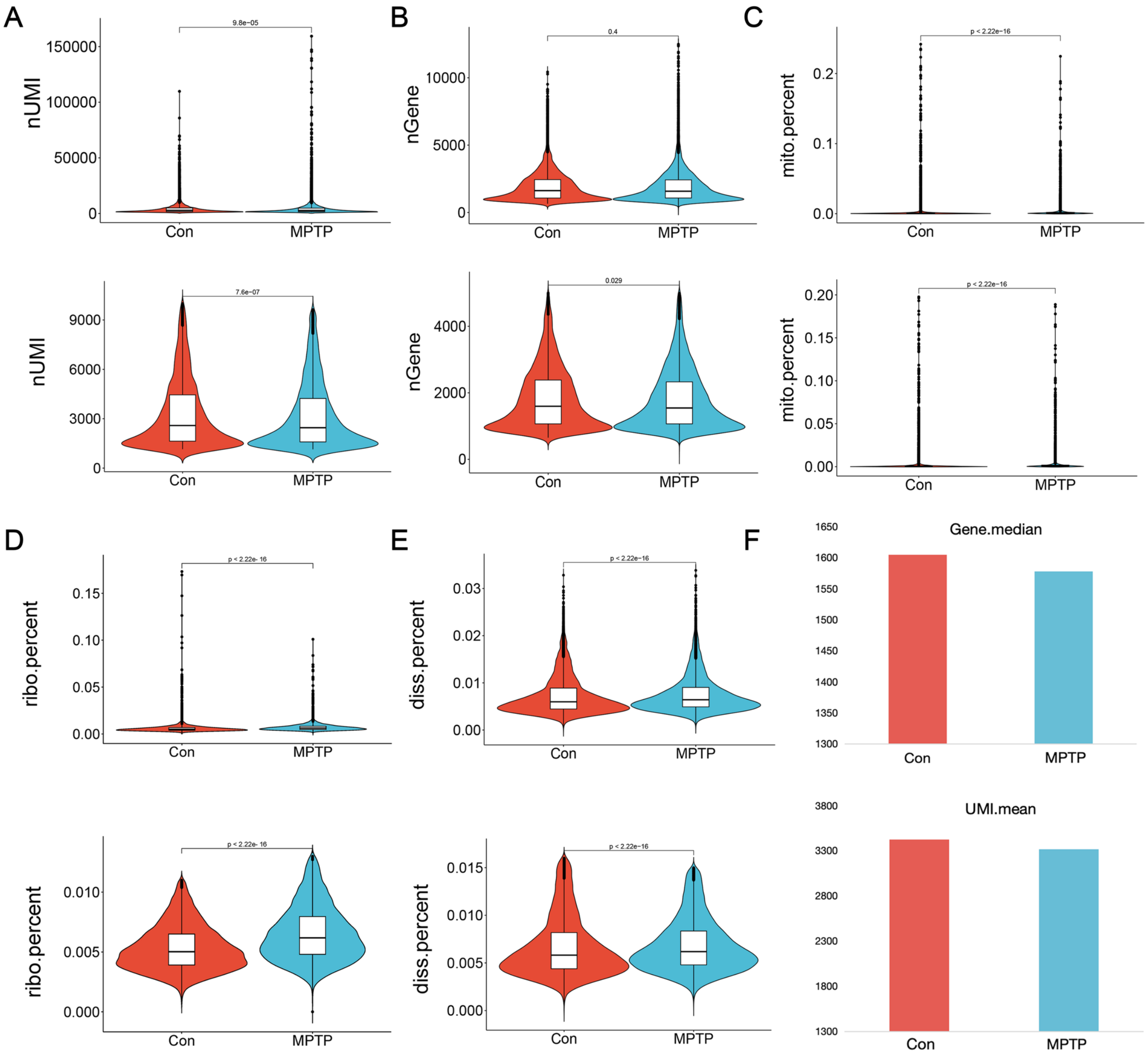

Supplementary Figure 2. snRNA-seq quality control metrics and similarity. (A) UMI number of samples before (top) and after (bottom) quality control. **(B)** Gene number of samples before (top) and after (bottom) quality control. **(C)** Mitochondrial gene percent of samples before (top) and after (bottom) quality control. **(D)** Ribosomal gene percent of samples before (top) and after (bottom) quality control. **(E)** Dissociated gene percent of samples before (top) and after (bottom) quality control. **(F)** Mean UMI and gene median for each sample.

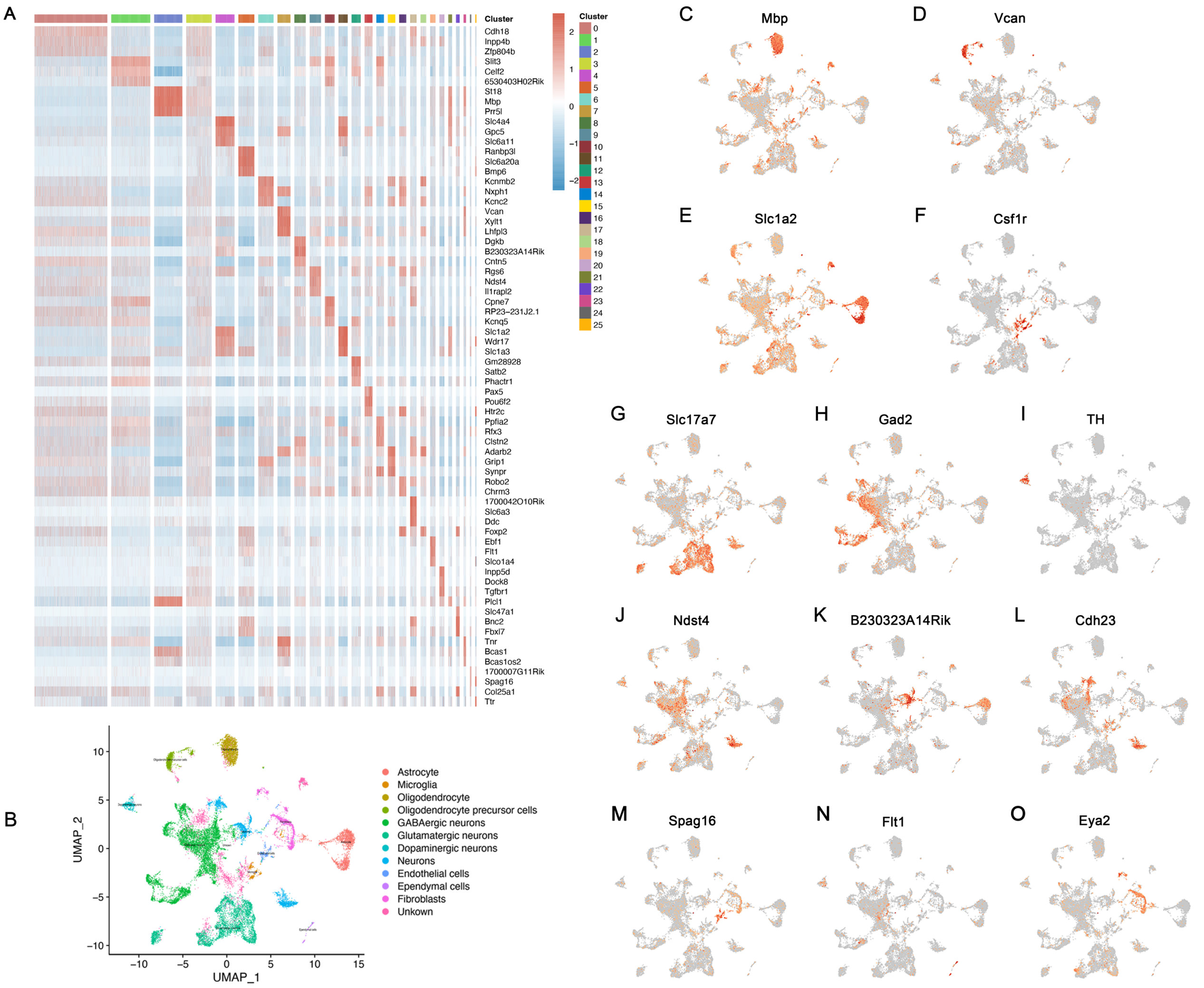

Supplementary Figure 3. Cell type marker genes. (A) Heat map of expression distribution of the top 3 marker genes in cell types. **(B)** UMAP embedding of the 22,983 mouse SN nuclei; colored by cell type. **(C)** Expression distribution of oligodendrocyte marker genes. **(D)** Expression distribution of OPC marker genes. **(E)** Expression distribution of astrocyte marker genes. **(F)** Expression distribution of microglial marker genes. **(G)** Expression distribution of Glu marker genes. **(H)** Expression distribution of GABA marker genes. **(I)** Expression distribution of DaNs marker genes. **(J-L)** Expression distribution of neuron marker genes. **(M)** Expression distribution of endothelial cell marker genes. **(N)** Expression distribution of ependymal cell marker genes. **(O)** Expression distribution of fibroblast marker genes.

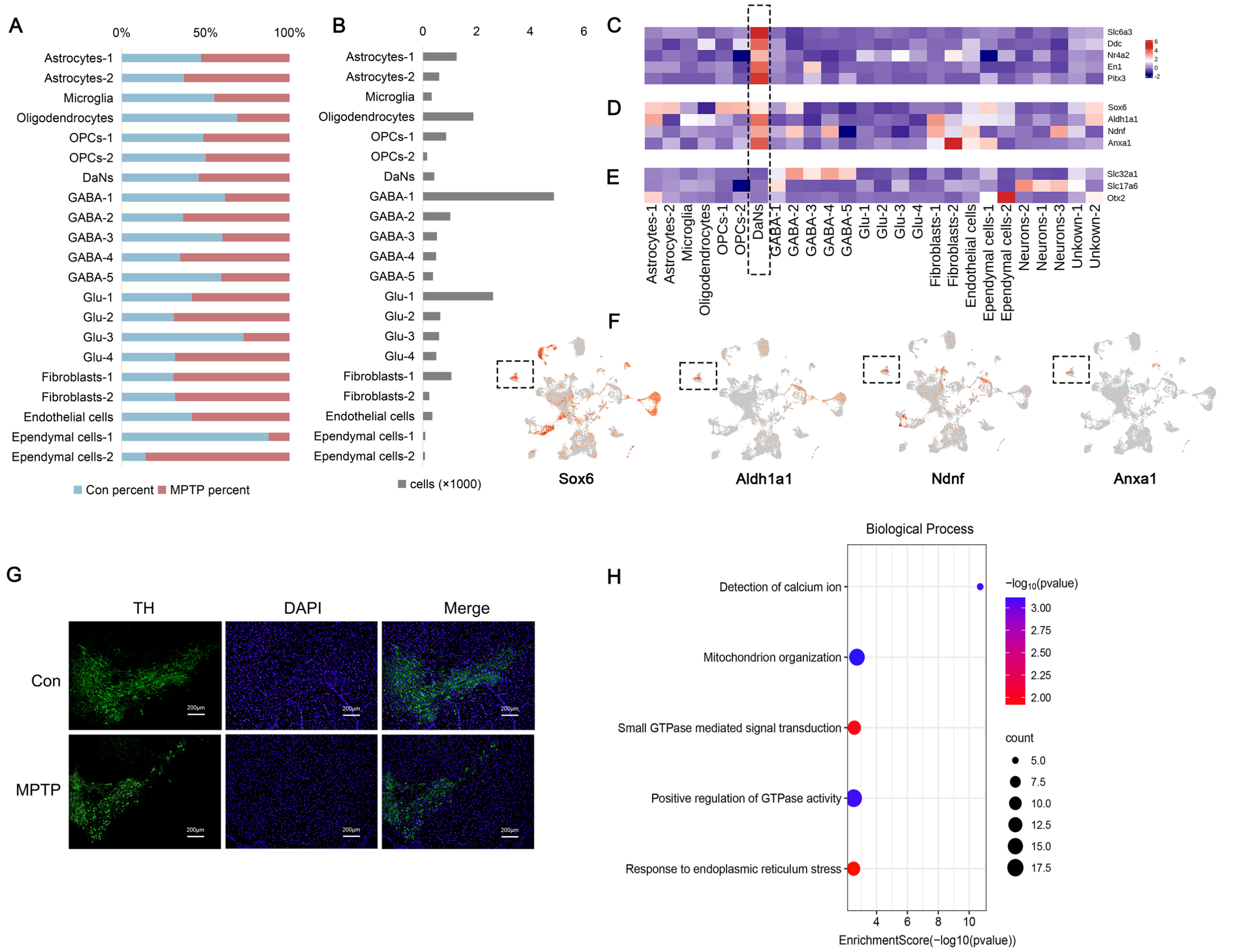

Supplementary Figure 4. About DaNs analysis. (A) Proportions of MPTP and Con cells for each cell type. (B) The number of nuclei per cell type. (C) Heat map of general marker genes in DaNs. (D) Heat map of marker genes in SN DaNs. (E) Heat map of marker genes in VTA DaNs. (F) UMAP of expression distribution of marker genes in SN DaNs. (G) The SN of MPTP and Con was immunofluorescent labeled with TH. (H) GO terms associated with DaNs DEGs.

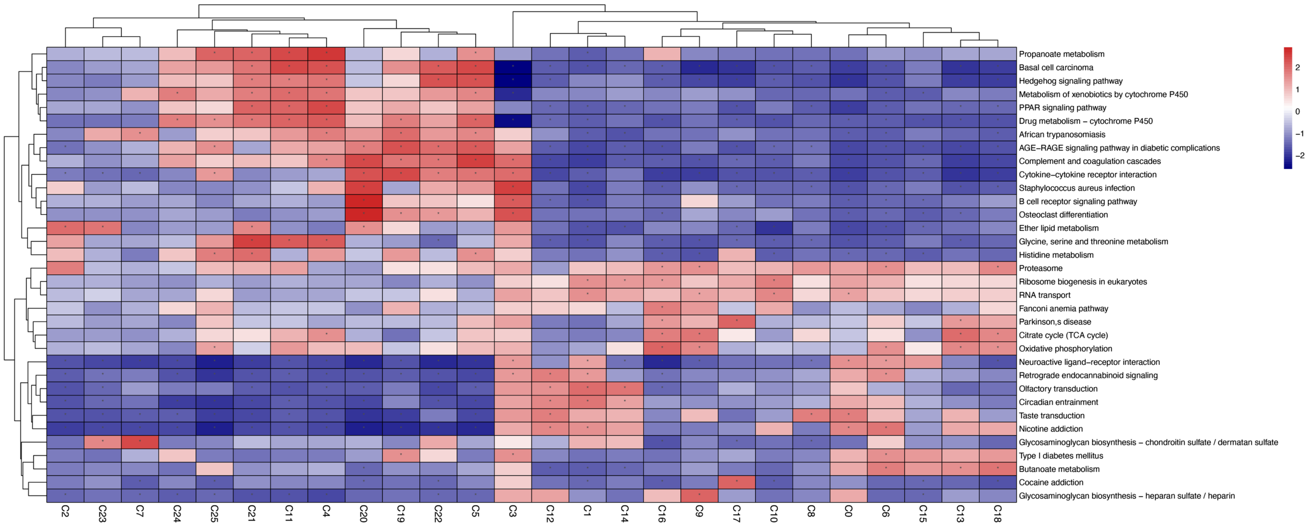

Supplementary Figure 5. Heatmap of GSEA results for each cluster maker gene.

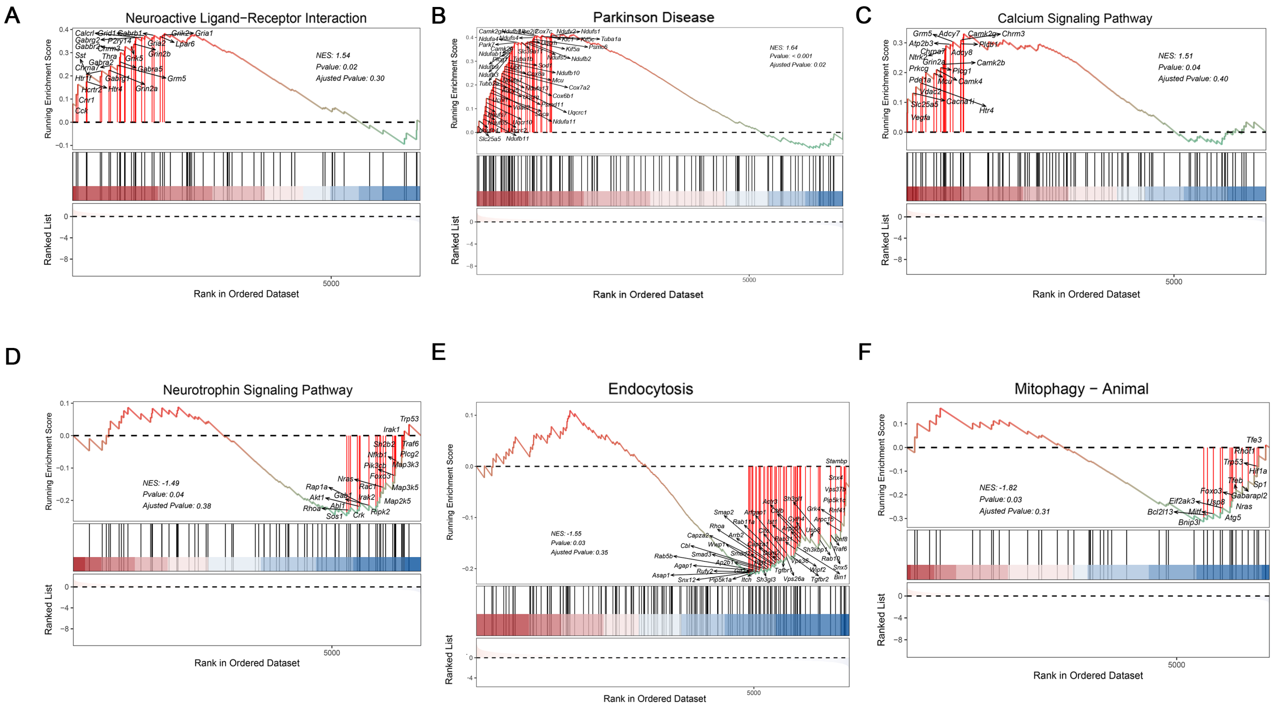

Supplementary Figure 6. Visualization of GSEA results for microglia DEGs. (A) Neuroactive ligand-receptor interaction. **(B)** Parkinson Disease. **(C)** Calcium signaling pathway. **(D)** Neurotrophin signaling pathway. **(E)** Endocytosis. **(F)** Mitophagy-animal. NES > 0, activated; NES < 0, inhibited.

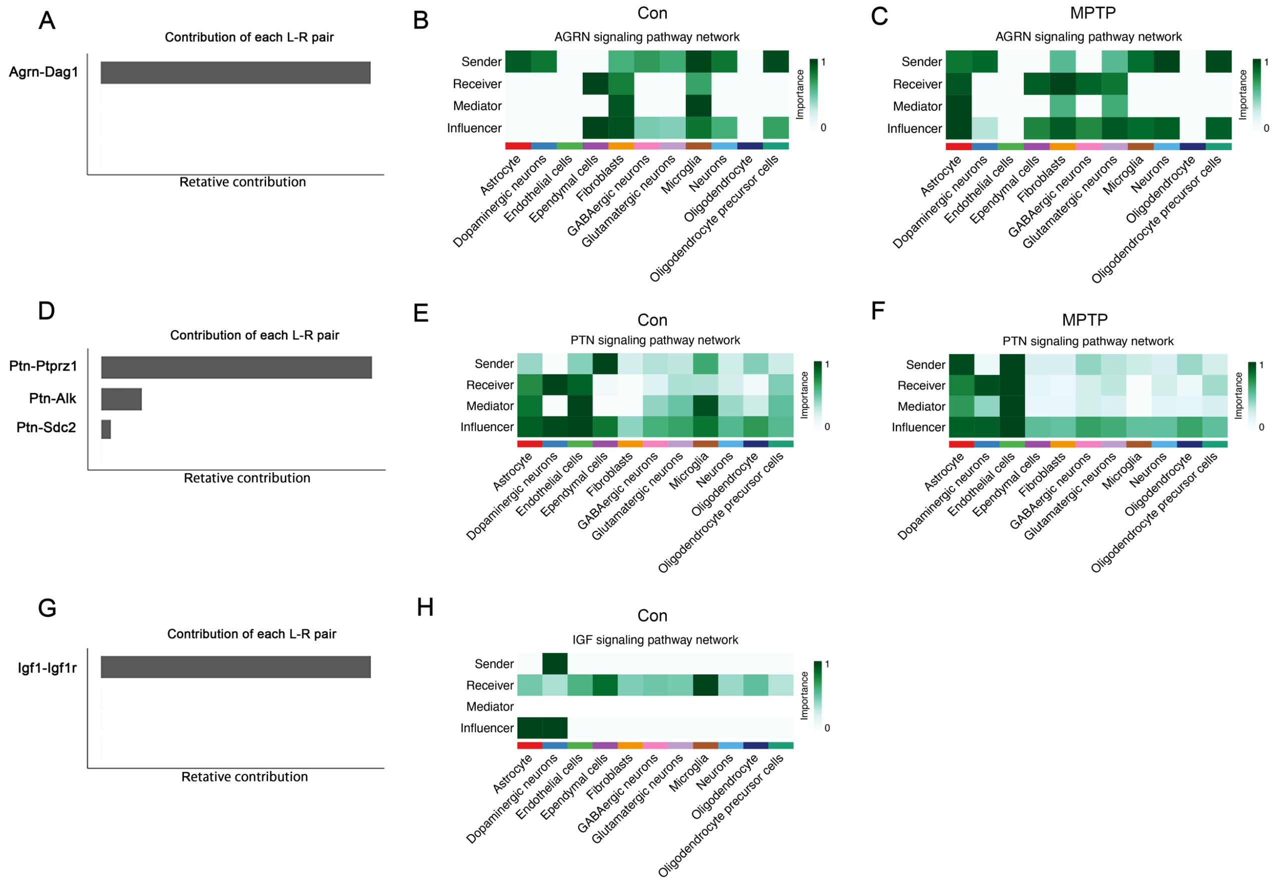

Supplementary Figure 7. L-R contributions and heatmaps of major signaling roles. (A) L-R contribution of IGF signaling. **(B)** Heatmap of IGF signaling pathway roles in Con. **(C)** L-R contribution of AGRN signaling. **(D)** Heatmap of AGRN signaling pathway roles in Con (left) and MPTP (right). **(E)** L-R contribution of PTN signaling. **(F)** Heatmap of PTN signaling pathway roles in Con (left) and MPTP (right).
